# An improved handmade snare-assisted method to optimize endoscopic tip angulation

**DOI:** 10.1055/a-2792-9950

**Published:** 2026-03-02

**Authors:** Tomona Sakurai, Haruhiro Inoue, Kazuki Yamamoto, Kei Ushikubo, Yohei Nishikawa, Ippei Tanaka, Mayo Tanabe

**Affiliations:** 1 378609 Digestive Diseases Center, Showa Medical University Koto Toyosu Hospital, Tokyo, Japan

Endoscopic treatment of lesions in the gastric cardia is technically challenging because of the limited maximal angulation range of the endoscope. To overcome this difficulty, we previously developed a handmade snare-assisted endoscope tip-bending angulation booster 1 . This device expands the effective bending range using a snare and suture, enabling precise procedures in anatomically difficult areas during endoscopic submucosal dissection and anti-reflux mucosal intervention (ARMI). However, the exposed metallic snare may directly contact the mucosa and potentially cause mechanical injury. Moreover, electrosurgical procedures such as monopolar dissection and argon plasma coagulation may induce unintended current conduction and thermal damage due to the adjacent metal 2 3 . Although more than 350 ARMI procedures using the original angulation booster have been performed at our institution without any complications, we aimed to further improve its safety. Therefore, we developed a modified device in which the snare is covered by an outer sheath to provide insulation and prevent potential mucosal injury.

**Fig. 1:**
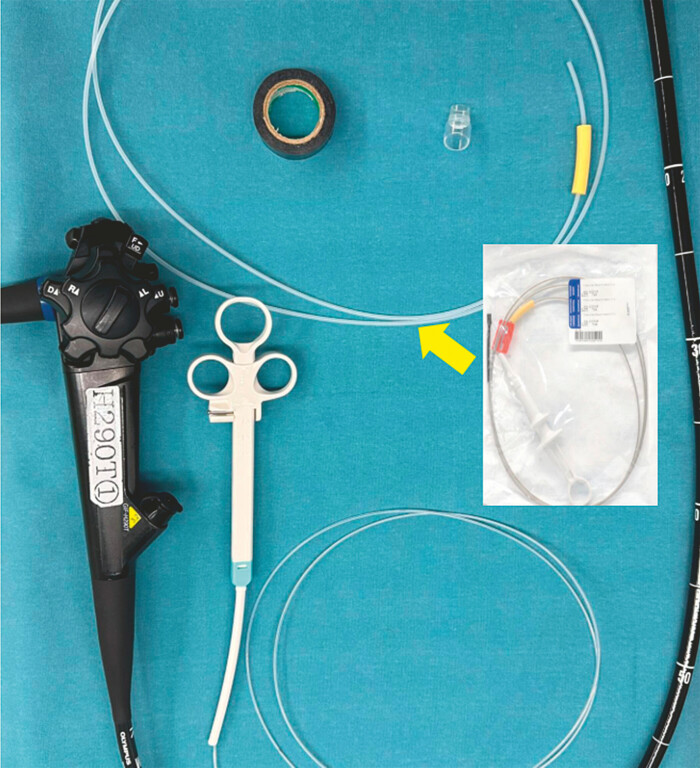
Preparation of materials.

**Fig. 2:**
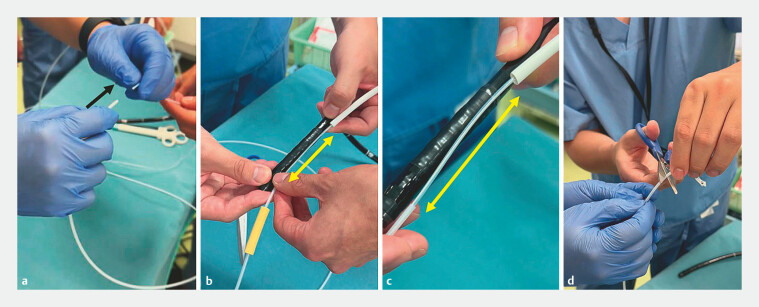
Construction of the improved angulation booster: Inserting the snare into the outer sheath and trimming the outer sheath. **a** The electrosurgical snare is inserted into the outer sheath. **b** The yellow arrow indicates the length from the proximal to the distal end of the endoscope’s bending section. **c** The proximal end of the snare is fixed approximately 10 cm from the tip of the endoscope, just proximal to the bending section. The sheath is advanced over the snare to the same position as the distal end of the bending section. **d** The sheath is precisely cut with scissors at the position corresponding to the distal end of the snare visible inside the sheath.

**Fig. 3:**
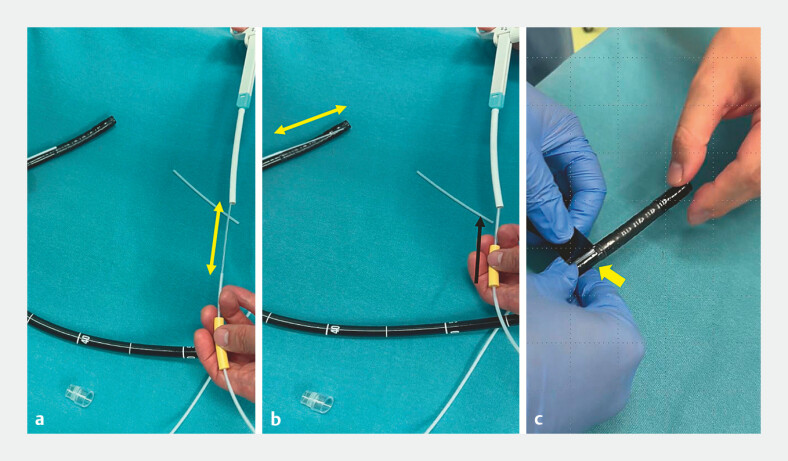
Construction of the improved angulation booster: Confirming snare movement and fixing the snare in the correct position. **a** The snare is housed within the sheath. **b** By pulling the outer sheath back along the snare, the snare extends out from the sheath. **c** The tip of the outer sheath is fixed with tape approximately 10 cm from the tip of the endoscope, just proximal to the bending section.

**Fig. 4:**
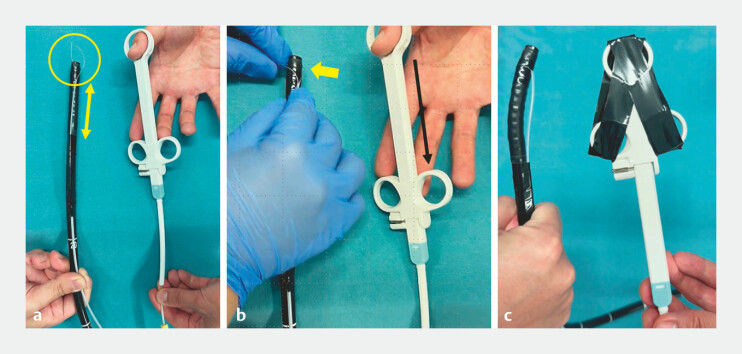
Construction of the improved angulation booster: Securing the snare. **a** The outer sheath is pulled back to expose the snare body and then the snare loop is opened. **b** The snare is used to grasp the section of the endoscopic tip that was previously covered with vinyl tape. **c** Once the snare securely grasps the taped area, the handle of the snare is secured with vinyl tape to prevent any movement during the procedure.

**Fig. 5:**
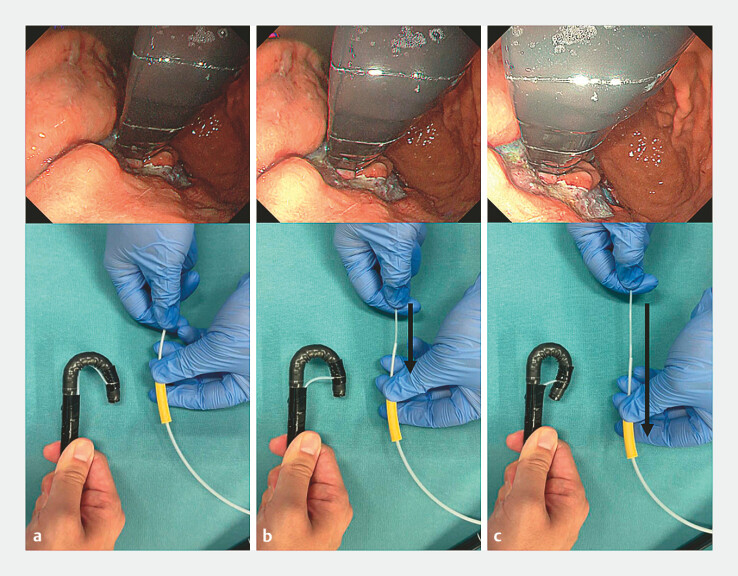
Manipulation of the improved angulation booster and the resulting changes in endoscopic tip angulation. **a** The maximum up-angle position. **b** The maximum up-angle position with the sheath pushed halfway forward. **c** The maximum up-angle position with the sheath pushed fully forward.

The improved angulation booster is constructed using an endoscopic snare, vinyl tape, and an outer sheath ( Fig. 1 ). The snare is inserted into the sheath, and the sheath is cut so that its length is shorter than the total length of the snare by the same distance as the bending section of the endoscope ( Fig. 2 ). The sheath is then fixed with tape at the proximal end of the bending section of the endoscope ( Fig. 3 ). After opening the snare loop to grasp the distal end of the endoscope, the area to be grasped is protected with tape ( Fig. 4 ). Pulling the outer sheath retracts the snare, improving upward angulation ( Fig. 5 and Video 1 ).

The video presents an improved handmade snare-assisted technique for endoscope tip angulation optimization. The need for device refinement, the safety-oriented modifications, the construction process, and its effective use in a clinical case are demonstrated.Video 1

The improved angulation booster is a simple, reproducible, and safer modification of the original device that facilitates secure endoscopic manipulation in difficult anatomical locations.

Endoscopy_UCTN_Code_TTT_1AO_2AD
